# Investigation of immunosuppressive treatment compliance, dyspnea, anxiety, and depression levels in lung transplant recipients: online interview

**DOI:** 10.3389/fpsyg.2024.1378594

**Published:** 2024-10-22

**Authors:** Semra Bulbuloglu, Halil Ibrahim Sayim

**Affiliations:** ^1^Division of Surgical Nursing, Nursing Department, Health Sciences Faculty, Istanbul Aydin University, Istanbul, Türkiye; ^2^Division of Surgical Nursing, Nursing Department, Health Sciences Faculty, Istanbul Arel University, Istanbul, Türkiye

**Keywords:** anxiety, immunosuppressive treatment compliance, depression, dyspnea, lung transplant recipients

## Abstract

**Introduction:**

Following lung transplantation (LTx), it is important for recipients to comply with immunosuppressive treatment and cope with related problems. In the post-LTx period, the course of dyspnea and psychological problems it causes in case of progression are not known. Depression and anxiety may develop in recipients after LTx. However, the relationship between this situation and treatment compliance and dyspnea is uncertain.

**Objective:**

The aim of this study was to investigate dyspnea, anxiety, and depression levels of recipients following LTx and their immunosuppressive treatment compliance.

**Method:**

The study was planned as a descriptive, correlational, and cross-sectional study. Data were collected on various social media platforms via an online interview, and 65 LTx recipients were included in the sample (*n* = 65). A Participant Information Form, the Modified Borg Scale, the Beck Anxiety Inventory, the Beck Depression Inventory, and the Immunosuppressive Drug Compliance Scale were employed to collect data. The collected data were analyzed using descriptive statistics, the Mann–Whitney U test, and the Kruskal-Wallis test.

**Results:**

The mean age of the participants was found to be 52.60 ± 9.44 years, and 56.9% were male. Forty percent of the participants were dependent on oxygen support, and 32.2% had hypertension. Their dyspnea levels were mild, anxiety levels were moderate, depression levels were high, and immunosuppressive treatment compliance levels were slightly above-average. According to the correlation analysis results, dyspnea was associated with anxiety and depression (*p* < 0.05). As depression increased, immunosuppressive treatment compliance decreased, and the correlation between the two variables was statistically significant (*p* < 0.05).

**Conclusion:**

In this study, as the severity of dyspnea experienced by LTRs increased, the severity of their anxiety and depression also increased. Additionally, there was an inverse correlation between depression and immunosuppressive treatment compliance. LTRs demonstrated insufficient adherence to their immunosuppressive drug regimens, which is very significant in terms of graft survivability. These results suggest that LTRs should be closely monitored at home.

## Introduction

Lung transplantation (LTx) is a life-saving surgical treatment option in terminal-stage respiratory failure (Scheffert and Raza, 2014). Post-LTx survival rates have been reported as 89.1% in the first 3 months, 80% up to a year, 65% in 3 years, 54% in 5 years, and 32% in 10 years (Yusen et al., 2016). Additionally, the median survival duration of LTx recipients (LTRs) was determined to be a little longer than 6 years (Chambers et al., 2018; Yusen et al., 2014). It is clear that LTx provides significant benefits for LTRs in terms of survival. However, the development of chronic lung allograft dysfunction (CLAD) cannot be prevented today in approximately 50% of LTRs (Thabut and Mal, 2017), and the reason for this outcome is still unknown. The cessation of immunosuppressive treatment by LTRs who may think they have completely recovered can be effective in CLAD development. In the literature, there are several studies in which immunosuppressive treatment drug regimen compliance in LTRs has been examined. Organ recipients are supposed to use immunosuppressive drugs at high dosages in the first 2 years and at lower dosages in the following years throughout their lives (Cajanding, 2018a, 2018b).

Despite a distinct improvement in pulmonary functions following LTx (Bartels et al., 2011), a restriction was determined in exercise capacity due to peripheral muscle dysfunction (Braccioni et al., 2020). In contrast, a 1.5- to 2-fold increase is expected in exercise capacity after LTx. In LTRs, postoperative increased forced vital capacity (FVC) and increased forced expiratory volume in the 1st second (FEV1) play an active role in the improvement of pulmonary functions. Nevertheless, increased oxygen consumption and high carbon dioxide production constitute a risk for the exacerbation of pulmonary functions (Bartels et al., 2011). All these problems prepare the ground for LTRs to encounter dyspnea.

Dyspnea develops as a complication in LTRs, and it has various physiological and psychological effects. Anxiety and depression are prominent psychological disorders in chronic lung diseases (Dew et al., 2012; Dew et al., 2023). In a limited number of articles, depression and anxiety have been reported to be the most frequently experienced psychiatric problems (approximately in 30% of recipients) after LTx (Corbett et al., 2013; Corruble et al., 2011; Dew et al., 2012). Depression was associated with mortality and graft loss (Rosenberger et al., 2016). In previous studies, researchers have mostly followed up patients for the first few years following LTx. Therefore, long-term results are quite limited, and the evidence is poor. One of the most important symptoms indicating the exacerbation of pulmonary functions is dyspnea, and as the severity of dyspnea increases, the likelihood of negative emotions may emerge in LTRs. Additionally, this situation may disrupt treatment compliance in LTRs. The objective of this study is (i) to measure the severity of dyspnea experienced by LTRs, (ii) determine the anxiety and depression levels of LTRs, and (iii) investigate immunosuppressive treatment compliance in LTRs.

## Materials and methods

This study was conducted with a descriptive, correlational, and cross-sectional design to investigate dyspnea, anxiety, and depression levels and immunosuppressive treatment compliance in LTRs.

### Study design and participants

The study was conducted with the participation of 65 LTRs (*n* = 65). Following ethics committee approval, the study was conducted with LTRs who underwent LTx and agreed to be included in the sample of the study. Data collection tools were applied by the researchers on social media platforms via an online interview, between 1 February and 12 July 2023. LTRs residing in or outside Turkey were contacted via social media accounts, e-mail addresses, and other electronic communication applications, and the link for the online data collection form was shared with them. LTRs gave their consent online, and they responded to the questions on the data collection tools online. No sampling was performed for the study, and those who met the inclusion criteria were included in the sample.

### Inclusion and exclusion criteria

Individuals at the age of 18 years and above who (i) had undergone LTx at least 6 weeks ago, (ii) had been discharged from the hospital, (iii) were literate in Turkish and had no communication and language barriers, and (iv) agreed to participate in the study and filled out the informed consent form were included in the sample. Those who did not meet the inclusion criteria were excluded from the study.

### Data collection tools

In the data collection process, a Participant Information From, the Modified Borg Scale, the Beck Anxiety Inventory, the Beck Depression Inventory, and the Immunosuppressive Drug Compliance Scale were employed.

### Participant Information Form

The form consisted of 13 questions designed to collect information about the sociodemographic and health-related characteristics of the participants including education levels, economic status, marital status, smoking status, oxygen support requirements, immunosuppressive drug and spirometer use status, age, and gender.

### Modified Borg Scale

The scale was developed by Borg (1982) to measure dyspnea severity during physical exercise. MBS consists of 10 items that define dyspnea severity according to their degree (Burdon et al., 1982). In dyspnea classification, “0” shows the absence of dyspnea, while “10” indicates the most severe degree of dyspnea experienced. In previous studies, it has been reported that MBS is a reliable scale in determining exercise and rest dyspnea severity, and it is correlated with respiratory rate and respiratory function tests (Akkoca et al., 2001; Grant et al., 1999).

### Beck Anxiety Inventory

The scale was developed by Beck et al. (1988). The Turkish adaptation study of the scale, in which its validity and reliability were tested, was conducted by Ulusoy et al. (1998). The 21-item 4-point Likert-type scale is a self-report scale that aims to measure the frequency of anxiety symptoms experienced by the individual. Responses to the scale items are scored as 0-Never, 1-Mildly, 2-Moderately, and 3-Severely. The minimum and maximum scores to be obtained from the scale are 0 and 63. A score of 8–15 indicates mild anxiety, a score of 16–25 indicates moderate anxiety, and a score of 26–63 indicates severe anxiety. The Cronbach’s alpha coefficient of the scale was calculated as 0.93 by Ulusoy et al. In this study, the Cronbach’s alpha coefficient of the scale was found to be 0.81.

### Beck Depression Inventory

The scale developed by Beck et al. (1961) consists of 21 items, which aim to measure the symptoms emerging in depression. Hisli (1988) created the Turkish form of the scale. In the assessment, parameters such as anhedonia, depressive moods, pessimism, sleep disorders, feelings of failure, weight loss, a distorted body image, crying fits, feelings of guilt, loss of appetite, irritability, social withdrawal, indecision, working inhibition, loss of libido, and somatic endeavors are used. The respondent chooses one of the four statements to indicate their psychological status at that moment. Each item is scored between 0 and 3. The highest score to be obtained is 63, and the lowest score is 0. A score of 17 or above on BDI indicates the presence of depression. Hisli found the Cronbach’s alpha coefficient of the scale as 0.80. This coefficient was determined to be 0.83 in this study.

### Immunosuppressive drug use compliance scale

The scale was developed by Özdemir Köken et al. (2019) to evaluate immunosuppressive drug use compliance in patients who have undergonesolid organ transplantation. The 11-item scale has one dimension, and it is scored using 5-point and 2-point Likert-type scoring systems. In the items scored via a 5-point system, positive statements are scored from 1 to 5, while negative statements are scored from 5 to 1. In the items requiring a response of “Yes-No,” a response of “yes” is given 1 point, while a response of “no” is given 5 points. The minimum and maximum scores to be obtained from the scale are 11 and 55, and high scores show a high level of compliance of the individual with immunosuppressive drug use.

### Statistical analysis

In the first stage of the analysis, the data were coded in the Excel software by the researchers. Then, the coded data were transferred from Excel to the Statistical Package for the Social Sciences 27.0 IBM (Armonk, NY) software. The normality of the distribution of the data was determined using the Kolmogorov–Smirnov test. In the analyses, descriptive statistics (percentage, mean, and standard deviation) were used. The relationships between the variables were examined with the Mann–Whitney U, Kruskal-Wallis, and Spearman’s Rho correlation analyses and *post hoc* Bonferroni tests. The reliability levels of the scales were determined using Cronbach’s alpha internal consistency coefficients. The data were analyzed within a 95% confidence interval and at a *p* < 0.05 significance level.

### Ethical aspect of the study

Prior to data collection, the necessary approval was obtained from Istanbul Arel University Ethics Committee (Date: 30.12.2022, Decision No: 2022/26/04). In line with the Declaration of Helsinki, written informed consent was obtained from LTRs who agreed to participate. Accordingly, LTRs who agreed to participate in the study had to tick the consent form in the electronic environment before they could move on to the questions.

## Results

Table 1 presents the individual characteristics of the participants. The mean age of the participants was 52.60 ± 9.44 years, 56.9% of them were male, 67.7% had moderate economic status, 52.3% were high school graduates, and 80% were married. The results showed that 36.9% of the participants had a history of smoking for 5–10 years, and 4.6% continued to smoke after their LTx. Oxygen support dependency was found in 40% of the participants, and 64.6% used the TriFlo^®^ II Incentive Spirometer. Hypertension was seen in 32.2% of the participants, and 21.5% had diabetes mellitus (DM). In this study 35.4% of LTRs used Tacrolimus + Mycophenolic Acid (MFA) + Corticosteroid treatment. According to the responses of the participants, 7.7% had stopped using their immunosuppressive drugs without consulting a doctor.

**Table 1:** Individual characteristics of the LTRs (*n* = 65).

Characteristics	x̄ ± Sd	Min–Max
Age	52.60 ± 9.44	24–73
	***n***	**%**
Gender		
Female	28	43.1
Male	37	56.9
**Marital status**		
Single	13	20.0
Married	52	80.0
**Economic status**		
Poor	6	9.2
Moderate	44	67.7
Good	15	23.1
**Education**		
Elementary school	17	26.2
High school	34	52.3
University and above	14	21.5
**Time of lung transplant**		
6 months ago	4	6.2
Between 6 months and 1 year ago	8	12.3
Between 1 and 2 years ago	23	35.4
Between 2 and 5 years ago	23	35.4
More than 5 years ago	7	10.8
**Smoking history**		
Never	6	9.2
Less than 5 years	16	24.6
Between 5 and 10 years	24	36.9
More than 10 years	19	29.2
**Current smoking status**		
Yes	3	4.6
No	62	95.4
**TriFlo® II incentive spirometer use**		
Yes	42	64.6
No	23	35.4
**Oxygen dependence**		
Yes	26	40.0
No	39	60.0
**Comorbid diseases**^a^		
Diabetes mellitus	14	21.5
Heart failure	4	6.2
Hypertension	21	32.2
Chronic renal failure	2	3.1
Liver diseases	4	6.2
Thyroid disorders	5	7.7
None	35	53.8
**Pulmonary problems**^**a**^		
Dyspnea	37	56.9
Wheezing + coughing	44	67.7
**Immunosuppressive drug used**		
MFA + corticosteroid	9	13.8
Calcineurin inhibitor + corticosteroid + tacrolimus	7	10.8
Tacrolimus + MFA + corticosteroid	23	35.4
Tacrolimus + calcineurin inhibitor + MFA + kortikosteroid	21	32.3
**Immunosuppressive use**		
Regular	49	75.4
Irregular	11	16.9
Quit without consulting a doctor	5	7.7

a Can have more than one options; MFA, Mycophenolic Acid.

Table 2 presents the mean scale scores of the participants. The mean IDUCS, BAI, BDI, and MBS scores of the participants were determined to be 26.73 ± 8.68, 25.64 ± 9.36, 35.92 ± 9.75, and 2.45 ± 2.12, respectively.

**Table 2:** LTRs’ mean scores on IDUCS, BAI, BDI, and dyspnea scale (*n* = 65).

Total scale and subscales	Number of items	Items	Score range	Min.–Max.	x̄ ± SD
IDUCS total	11	Items 1*, 2*, 3*, 4, 5*, 6, 7*, 8*, 9, 10, and 11	11–55	11–46	26.73 ± 8.68
BAI total	21	Items 1–21	0–63	0–43	25.64 ± 9.36
BDI total	21	Items 1–21	0–63	21–61	35.92 ± 9.75
Dyspnea scale total	1	Item 1	0–10	0–10	2.45 ± 2.12

*Reversely coded items.

Table 3 shows the results of the comparisons of the IDUCS, BAI, BDI, and MBS scores of the participants based on some of their characteristics. A negative, weak, and significant relationship was found between age and immunosuppressive treatment compliance (*p* = 0.047). There was a positive, moderate, and significant relationship between age and depression levels (*p* = 0.006). The female participants had significantly higher BAI, BDI, and MBS scores than the male participants (*p* < 0.05). In comparison to the participants who had undergone LTx more than 2 years ago, those who had undergone LTx less than 2 years ago had significantly higher IDUCS, BAI, BDI, and MBS scores (*p* < 0.05). The participants who were smokers and those who were using the TriFlo^®^ II Incentive Spirometer had significantly higher BDI and MBS scores (*p* < 0.05). The participants who were dependent on oxygen support had significantly higher BAI, BDI, and MBS scores (*p* < 0.05).

**Table 3:** Comparison of some individual variables of LTRs with IDUCS, BAI, BDI, and dyspnea scale (*n* = 65).

Characteristics	x̄±Sd	Min–Max	IDUCS	BAI	BDI	Dyspnea scale
**Age**	52.60 ± 9.44	24–73	26.73 ± 8.68	25.64 ± 9.36	35.92 ± 9.75	2.45 ± 2.12
			*r* = −0.207, *p* = 0.047*	–	*r* = 0.481, *p* = 0.006**	–
	***n***	**%**				
**Gender**						
Female	28	43.1	27.32 ± 7.89	16.21 ± 7.83	36.42 ± 11.11	2.51 ± 0.74
Male	37	56.9	26.29 ± 7.32	15.21 ± 7.24	35.54 ± 8.72	2.40 ± 0.40
**Test and sig.**			*U* = 0.231, *p* = 0.061	*U* = 0.359, *p* = 0.025*	*U* = 0.416, *p* = 0.028*	*U* = 1.049, *p* = 0.035*
**Time of lung transplant**						
6 months ago (1)	4	6.2	29.13 ± 7.85	18.75 ± 6.25	37.38 ± 8.24	3.08 ± 0.82
Between 6 months and 1 year ago (2)	8	12.3	27.31 ± 7.28	19.12 ± 7.81	39.38 ± 6.91	3.87 ± 0.97
Between 1 and 2 years ago (3)	23	35.4	27.02 ± 7.54	17.69 ± 6.65	38.74 ± 7.35	2.67 ± 0.95
Between 2 and 5 years ago (4)	23	35.4	26.73 ± 7.38	13.86 ± 9.14	34.07 ± 8.5	1.91 ± 0.57
More than 5 years ago (5)	7	10.8	25.29 ± 6.91	11.14 ± 7.43	33.43 ± 8.49	1.57 ± 0.91
**Test and sig.**			KW = 0.955, *p* = 0.035*	KW = 0.753, *p* = 0.016*	KW = 1.502, *p* = 0.047*	KW = 0.845, *p* = 0.014*
***Post hoc* Bonferroni**			1,2,3>4,5	1,2,3>,4,5	1,2,3>,4,5	1,2,3>4,5
**Current smoking status**						
Yes	3	4.6	30 ± 5	26 ± 8	44.33 ± 6.50	2.49 ± 1.99
No	62	95.4	26.58 ± 8.82	15.14 ± 9.24	35.51 ± 8.73	2.06 ± 1.15
**Test and sig.**			*U* = 0.840, *p* = 0.424	*U* = 1.646, *p* = 0.052	*U* = 0.987, *p* = 0.044*	*U* = 0.621, *p* = 0.046*
**TriFlo® II incentive spirometer use**						
Yes	42	64.6	27.04 ± 8.94	16.92 ± 7.96	37.28 ± 9.43	2.72 ± 1.35
No	23	35.4	26.17 ± 8.37	13.30 ± 7.88	33.43 ± 9.03	1.91 ± 1.45
			*U* = 0.426, *p* = 0.670	*U* = 0.623, *p* = 0.105	*U* = 1.596, *p* = 0.011*	*U* = 1.601, *p* = 0.009**
**Oxygen dependence**						
Yes	26	40.0	27.51 ± 8.50	20.73 ± 8.75	40.69 ± 9.22	3.25 ± 1.97
No	39	60.0	25.57 ± 8.83	12.75 ± 8.42	32.74 ± 8.84	1.92 ± 1.38
			*U* = 0.932, *p* = 0.351	*U* = 0.256, *p* = 0.001**	*U* = 0.311, *p* = 0.001**	*U* = 0.426, *p* = 0.001**

U, Mann Whitney U Test, KW, Kruskal Wallis Test, r, Spearman’s correlation test; *p < 0.05, **p < 0.01. Bold values indicate statistically significant.

The results of the correlation analyses of the IDUCS, BAI, BDI, and MBS scores of the participants are presented in Table 4. No statistically significant relationship was found between dyspnea severity and compliance with immunosuppressive treatment (*p* > 0.05). There was also no significant relationship between compliance with immunosuppressive treatment and anxiety levels (*p* > 0.05). A negative, moderate, and significant relationship was identified between compliance with immunosuppressive treatment and depression levels (*p* = 0.007). Positive, strong, and significant relationships were observed between dyspnea severity and anxiety levels (*p* = 0.016) and between dyspnea severity and depression levels (0.017).

**Table 4:** Correlation analysis between IDUCS, BAI, BDI, and dyspnea scale (*n* = 65).

	Items		Immunosuppressive treatment compliance	Dyspnea
Spearman's rho	Dyspnea	Correlation coefficient	0.134	–
		Sig. (2-tailed)	0.288	
		*N*	65	
	Depression	Correlation coefficient	–0.462	0.296
		Sig. (2-tailed)	0.007**	0.017*
		*N*	65	65
	Anxiety	Correlation coefficient	–0.360	0.298
		Sig. (2-tailed)	0.092	0.016*
		*N*	65	65

*Correlation is significant at the 0.05 level (2-tailed).

**Correlation is significant at the 0.01 level (2-tailed).

Bold values indicate statistically significant.

## Discussion

LTRs face psychiatric problems for the first time in the postoperative period. Facing the risk of death, being unable to find a donor, and the possibility of surgical failure in case of finding a donor may cause candidates of LTx to be exposed to high levels of stress, which can result in deterioration in their mental health. The results showed that LTRs experienced moderate levels of anxiety and high levels of depression. The previous studies reported that significant neuropsychiatric symptoms, including depression and anxiety, were observed in LTx candidates (Limbos et al., 2000; Parekh et al., 2003), and the incidence of psychiatric disorders in LTx candidates was 25% (Parekh et al., 2003). Frequently observed psychiatric problems observed in some LTx candidates continue to be experienced after LTx (Wessels-Bakker et al., 2022).

In a previous study, depressive symptoms that persist after LTx in LTRs were associated with mortality (Smith et al., 2014). Similarly, as postoperative anxiety levels in LTRs increased, their rates of physiological problems also increased (Dabbs et al., 2003). Several studies in the literature have shown that depressive symptoms and high levels of feelings of anger-hostility increase the risk of graft rejection in the first 3 years following a heart transplant (Rogal et al., 2013), and depressive symptoms trigger mortality in liver transplant recipients (Dew et al., 1999). Considering the physiological problems resulting from psychiatric problems reported in the literature, the importance of the treatment of depression and anxiety experienced by LTRs in the shortest time possible is clear.

Psychiatric problems experienced by LTx candidates and LTRs and the physiological outcomes of these problems have been analyzed in a limited number of studies in the literature. Although dyspnea appears to be an inevitable symptom in patients with asthma and chronic obstructive pulmonary disease (COPD) (Livermore et al., 2008), it is believed that both state anxiety and trait anxiety are associated with dyspnea development in healthy individuals (Herzog et al., 2018; Sharma et al., 2016). What is worth noting is that high levels of anxiety and depression are seen frequently in dyspneic individuals (Willgoss and Yohannes, 2013), or in contrast, the fear of suffocation and the feeling of breathlessness may lead to depression and anxiety. In this study, dyspnea severity was determined to have separate positive, strong, and statistically significant relationships to anxiety and depression. According to these relationships, as the severity of dyspnea in the participants increased, the severity of their depression and that of their anxiety also increased. In the study conducted by Ricotti et al. (2006) with the participation of 129 LTRs (in postoperative 9th month), the prevalence of dyspnea was reported to be 39%. In this study, 65 LTRs had dyspnea. Of the LTRs included in the sample, 66.1% had undergone LTx more than 5 years ago. Comparing the data of the study by Ricotti et al. (2006) and the data obtained in this study, a longer time after LTx may have increased the severity of dyspnea in LTRs, and they may have quit care practices for healing respiratory functions thinking that they have completely recovered or experienced burnout over time.

Dyspnea is a subjective feeling of restriction and discomfort that occurs as a result of the disruption of the harmony between the afferent information obtained through receptors and the respiratory center and respiratory muscles (Parshall et al., 2012). In the intensive care unit, the prevalence of dyspnea in mechanically ventilated patients was 47% (Schmidt et al., 2011), in recipients was reported as 63% in the early post-LTx period (Sato et al., 2022). In a multicenter study conducted in Italy, the prevalence of dyspnea was recorded as 39% at 9 months after LTx (Ricotti et al., 2006). In our study, 70.8% of LTRs were in the post-LTx period of 1–5 years. The prevalence of dyspnea for the entire sample was 56.9%. None of the recipients in our study reported CLAD. In this study, the mean MBS score of the participants was determined to be 2.45 ± 2.12, indicating that they had mild-to-moderate levels of dyspnea. Dyspnea in LTRs may be related to gradually decreasing functional pulmonary capacity in long-term. In a study conducted with the participation of 21 COPD patients, the same scale as the one used in this study (MBS) was used, and the mean score of the patients was found to be 3.5 ± 2 [moderately to mildly severe] (İnce et al., 2011). Although dyspnea levels in LTRs are usually lower than those in COPD patients, these levels are still worth consideration. This is because in a study previously conducted on COPD patients, it was reported that as dyspnea severity increased, the fear of death also increased (Bülbüloğlu and Kaplan Serin, 2023). In this study, there were LTRs who received the maximum score on MBS, and the rate of those with oxygen support dependency was 40%. Not being able to resolve pulmonary problems after LTx may cause LTRs to experience disappointment. Additionally, just as in COPD patients (Bülbüloğlu and Kaplan Serin, 2023), this situation may cause LTRs to experience the fear of death. This lays the ground for dyspnea to trigger anxiety and depression.

The results showed that 24.6% of the participants did not use their immunosuppressive drugs regularly, and 7.7% quitted taking their medicine without consulting a doctor. The IDUCS scores of the participants were slightly above average. The participants of this study who had been receiving treatment for longer than 2 years had lower levels of adhering to their immunosuppressive treatment (*p* = 0.035). In some other studies, it has been determined that 21% of renal transplant recipients forget to take their immunosuppressive drugs from time to time (Goldfarb-Rumyantzev et al., 2010) and that 33% are late in taking their medicine on time (Burra et al., 2011). Liver transplant recipients were reported to have relatively high drug compliance levels (Bülbüloğlu and Demir, 2021). In a study conducted with patients within 2 years after LTx, it was determined that 13% of LTRs failed to comply with their immunosuppressive treatment (Dew et al., 2008). The results of this study mostly agreed with the results of other studies in the relevant literature.

In this study, 16.9% of the participants used their immunosuppressive drugs irregularly. LTRs who do not comply with immunosuppressive treatment are undoubtedly at risk of CLAD. There is limited evidence in the literature about compliance with immunosuppressive treatment in patients who have received long-term treatment. In this sense, the findings of this study are very important. However, this study had certain limitations, which included its small sample size, and the data collection tools were self-report scales, the data on some characteristics of the participants were based on their subjective thoughts and feelings. The other limitation principle is that the data were collected online rather than through face-to-face interviews. Therefore, participants were determined online whether they met the inclusion criteria for the sample. The participants were those who volunteered their time, and this may have led to a sample selection bias, which was a limitation. As another limitation, the pulmonary function testing and pre-LTx respiratory examination results of the participants were not analyzed in this study. This situation is related to the fact that it has not been determined whether there is a physiological source of dyspnea. Additionally, hypertension and heart failure, which may have been experienced by some participants, may have triggered dyspnea in them. The severity of depression and anxiety may have been increased by daily problems or situations related to the private lives of the participants.

## Conclusion

The immunosuppressive treatment compliance levels of recipients after LTx should be maintained at the maximum level to ensure graft survivability. LTRs and their relatives should continuously be informed and motivated to ensure that their immunosuppressive drug treatment is be maintained at varying doses throughout their lives. The finding in this study that women experienced higher levels of depression, anxiety, and dyspnea in comparison to men suggested that women need more support in this context. The awareness of physicians and nurses in this regard should be raised. The fact that dyspnea experienced by LTRs mostly continued at varying degrees of severity. This situation suggested that more respiratory exercises and interventions that relieve the lungs should be performed. There were also LTRs in this study who used spirometers as well as those who continued to smoke. LTRs should be periodically educated on lung health. As the severity of dyspnea increased, depression and anxiety levels increased as well. Hence, LTRs should be provided with psychological support and psychotherapy.

## Data Availability

The raw data supporting the conclusions of this article will be made available by the authors, without undue reservation.

## References

[ref1] Akkoca Ö. Öner F. Saryal S. (2001). The relationship between dyspnea and pulmonary functions, arterial blood gasses and exercise capacity İn patients with COPD. Tuberculosis Thoracic J. 49, 431–438.

[ref2] Bartels M. N. Armstrong H. F. Gerardo R. E. Layton A. M. Emmert-Aronson B. O. Sonett J. R.. (2011). Evaluation of pulmonary function and exercise performance by cardiopulmonary exercise testing before and after lung transplantation. Chest 140, 1604–1611. doi: 10.1378/chest.10-2721, PMID: 21680643

[ref3] Beck A. T. Epstein N. Brown G. Steer R. A. (1988). An inventory for measuring clinical anxiety: psychometric properties. J. Consult. Clin. Psychol. 56, 893–897. doi: 10.1037/0022-006X.56.6.893 3204199

[ref4] Beck A. T. Ward C. H. Mendelson M. Mock J. Erbaugh J. (1961). An inventory for measuring depression. Arch. Gen. Psychiatry 4, 561–571. doi: 10.1001/archpsyc.1961.01710120031004 13688369

[ref5] Borg G. A. V. (1982). Psychophysical basis of perceived exertion. Med. Sci. Sports Exerc. 14, 377–381. doi: 10.1249/00005768-198205000-00012 7154893

[ref6] Braccioni F. Bottigliengo D. Ermolao A. Schiavon M. Loy M. Marchi M. R.. (2020). Dyspnea, effort and muscle pain during exercise in lung transplant recipients: an analysis of their association with cardiopulmonary function parameters using machine learning. Respir. Res. 21, 1–11. doi: 10.1186/s12931-020-01535-5 33059678 PMC7559436

[ref7] Bülbüloğlu S. Demir B. (2021). The effect of perceived social support on psychological resilience in liver transplant patients receiving immunosuppression therapy. Transpl. Immunol. 69:101475. doi: 10.1016/j.trim.2021.101475, PMID: 34600070

[ref8] Bülbüloğlu S. Kaplan Serin E. (2023). Effect of perceived dyspnea on attitude toward death from the perspective of COPD patients. OMEGA 86, 913–929. doi: 10.1177/0030222821993629, PMID: 33567984

[ref9] Burdon J. G. Juniper E. F. Killian K. J. Hargreave F. E. Campbell E. J. (1982). The perception of breathlessness in asthma. Am. Rev. Respir. Dis. 126, 825–828, PMID: 7149447 10.1164/arrd.1982.126.5.825

[ref10] Burra P. Germani G. Gnoato F. Lazzaro S. Russo F. P. Cillo U.. (2011). Adherence in liver transplant recipients. Liver Transpl. 17, 760–770. doi: 10.1002/lt.22294, PMID: 21384527

[ref11] Cajanding R. (2018a). Immunosuppression following organ transplantation. Part 1: mechanisms and immunosuppressive agents. Br. J. Nurs. 27, 920–927. doi: 10.12968/bjon.2018.27.16.920, PMID: 30187798

[ref12] Cajanding R. (2018b). Immunosuppression following organ transplantation. Part 2: complications and their management. Br. J. Nurs. 27, 1059–1065. doi: 10.12968/bjon.2018.27.18.1059, PMID: 30281349

[ref13] Chambers D. C. Cherikh W. S. Goldfarb S. B. Hayes D. Kucheryavaya A. Y. Toll A. E.. (2018). The international thoracic organ transplant registry of the International Society for Heart and Lung Transplantation: thirty-fifth adult lung and heart-lung transplant report—2018; focus theme: multiorgan transplantation. J Heart Lung Transplant. 37, 1169–1183. doi: 10.1016/j.healun.2018.07.020 30293613

[ref14] Corbett C. Armstrong M. J. Parker R. Webb K. Neuberger J. M. (2013). Mental health disorders and solid-organ transplant recipients. Transplantation 96, 593–600. doi: 10.1097/TP.0b013e31829584e0 23743726

[ref15] Corruble E. Barry C. Varescon I. Falissard B. Castaing D. Samuel D. (2011). Depressive symptoms predict long-term mortality after liver transplantation. J. Psychosom. Res. 71, 32–37. doi: 10.1016/j.jpsychores.2010.12.008, PMID: 21665010

[ref16] Dabbs A. D. V. Dew M. A. Stilley C. S. Manzetti J. Zullo T. McCurry K. R.. (2003). Psychosocial vulnerability, physical symptoms and physical impairment after lung and heart–lung transplantation. J. Heart Lung Transplant. 22, 1268–1275. doi: 10.1016/S1053-2498(02)01227-5, PMID: 14585388

[ref17] Dew M. A. DiMartini A. F. Dabbs A. J. D. Fox K. R. Myaskovsky L. Posluszny D. M.. (2012). Onset and risk factors for anxiety and depression during the first 2 years after lung transplantation. Gen. Hosp. Psychiatry 34, 127–138. doi: 10.1016/j.genhosppsych.2011.11.009, PMID: 22245165 PMC3288337

[ref18] Dew M. A. DiMartini A. F. Dabbs A. D. V. Zomak R. De Geest S. Dobbels F.. (2008). Adherence to the medical regimen during the first two years after lung transplantation. Transplantation 85, 193–202. doi: 10.1097/TP.0b013e318160135f, PMID: 18212623 PMC3387784

[ref19] Dew M. A. DiMartini A. F. Posluszny D. M. (2023). “Impact of the transplantation process on the caregiver” in Transplant psychiatry: a case-based approach to clinical challenges (Cham: Springer International Publishing), 219–224.

[ref20] Dew M. A. Kormos R. L. Roth L. H. Murali S. DiMartini A. Griffith B. P. (1999). Early post-transplant medical compliance and mental health predict physical morbidity and mortality one to three years after heart transplantation. J. Heart Lung Transplant. 18, 549–562. doi: 10.1016/S1053-2498(98)00044-8, PMID: 10395353

[ref21] Goldfarb-Rumyantzev A. S. Wright S. Ragasa R. Ostler D. Van Orden J. Smith L.. (2010). Factors associated with nonadherence to medication in kidney transplant recipients. Nephron Clin. Pract. 117, c33–c39. doi: 10.1159/000319645 20689323

[ref22] Grant S. Aitchison T. Henderson E. A. (1999). A comparison of the reproducibility and the sensitivity to change of visual analogue scales, Borg scales, and Likert scales in Normal subjects during submaximal exercise. Chest 116, 1208–1217. doi: 10.1378/chest.116.5.1208, PMID: 10559077

[ref23] Herzog M. Sucec J. Van Diest I. Van den Bergh O. Chan P. Y. Davenport P.. (2018). Reduced neural gating of respiratory sensations is associated with increased dyspnoea perception. Eur. Respir. J. 52:1800559. doi: 10.1183/13993003.00559-2018 29773692

[ref24] Hisli N. (1988). A study of validity of beck depression inventory. Turk. J. Psychol. [Türk Psikoloji Derg] 6, 118–126.

[ref25] İnce D. İ. Savcı S. Sağlam M. Güçlü M. B. Arıkan H. Çöplü L. (2011). Relationship between smoking history and functional capacity in patients with chronic obstructive pulmonary disease. Physiother. Rehabil. 22, 39–43.

[ref26] Limbos M. M. Joyce D. P. Chan C. K. Kesten S. (2000). Psychological functioning and quality of life in lung transplant candidates and recipients. Chest 118, 408–416. doi: 10.1378/chest.118.2.408, PMID: 10936133

[ref27] Livermore N. Butler J. E. Sharpe L. McBain R. A. Gandevia S. C. McKenzie D. K. (2008). Panic attacks and perception of inspiratory resistive loads in chronic obstructive pulmonary disease. Am. J. Respir. Crit. Care Med. 178, 7–12. doi: 10.1164/rccm.200711-1700OC, PMID: 18436789

[ref28] Özdemir Köken Z. Talas M. S. Gökmen D. (2019). Development and psychometric testing of the Turkish immunosuppressive medication adherence scale. Turk. J. Nephrol. 28, 120–126. doi: 10.5152/turkjnephrol.2019.3371

[ref29] Parekh P. I. Blumenthal J. A. Babyak M. A. Merrill K. Carney R. M. Davis R. D.. (2003). Psychiatric disorder and quality of life in patients awaiting lung transplantation. Chest 124, 1682–1688. doi: 10.1378/chest.124.5.1682, PMID: 14605035

[ref30] Parshall M. B. Schwartzstein R. M. Adams L. Banzett R. B. Manning H. L. Bourbeau J.. (2012). An official American Thoracic Society statement: update on the mechanisms, assessment, and management of dyspnea. Am. J. Respir. Crit. Care Med. 185, 435–452. doi: 10.1164/rccm.201111-2042ST, PMID: 22336677 PMC5448624

[ref31] Ricotti S. Vitulo P. Petrucci L. Oggionni T. Klersy C. (2006). Determinants of quality of life after lung transplant: an Italian collaborative study. Monaldi Arch. Chest Dis. 65, 5–12. doi: 10.4081/monaldi.2006.579, PMID: 16700187

[ref32] Rogal S. S. Dew M. A. Fontes P. DiMartini A. F. (2013). Early treatment of depressive symptoms and long-term survival after liver transplantation. Am. J. Transplant. 13, 928–935. doi: 10.1111/ajt.12164, PMID: 23425326 PMC3618550

[ref33] Rosenberger E. M. DiMartini A. F. Dabbs A. J. D. Bermudez C. A. Pilewski J. M. Toyoda Y.. (2016). Psychiatric predictors of long-term transplant-related outcomes in lung transplant recipients. Transplantation 100, 239–247. doi: 10.1097/TP.0000000000000824, PMID: 26177087 PMC4684444

[ref34] Sato T. Tanaka S. Akazawa C. Tsuda Y. Teraguchi S. Kai S.. (2022). Provider-documented dyspnea in intensive care unit after lung transplantation. Transplant. Proc. 54, 2337–2343. doi: 10.1016/j.transproceed.2022.08.034, PMID: 36180255

[ref35] Scheffert J. L. Raza K. (2014). Immunosuppression in lung transplantation. J. Thorac. Dis. 6:1039. doi: 10.3978/j.issn.2072-1439.2014.04.23 25132971 PMC4133546

[ref36] Schmidt M. Demoule A. Polito A. Porchet R. Aboab J. Siami S.. (2011). Dyspnea in mechanically ventilated critically ill patients. Crit. Care Med. 39, 2059–2065. doi: 10.1097/CCM.0b013e31821e8779 21572329

[ref37] Sharma P. Morris N. R. Adams L. (2016). Effect of experimental modulation of mood on perception of exertional dyspnea in healthy subjects. J. Appl. Physiol. 120, 114–120. doi: 10.1152/japplphysiol.00122.2015, PMID: 26565017

[ref38] Smith P. J. Blumenthal J. A. Carney R. M. Freedland K. E. O'Hayer C. V. F. Trulock E. P.. (2014). Neurobehavioral functioning and survival following lung transplantation. Chest 145, 604–611. doi: 10.1378/chest.12-2127, PMID: 24233282 PMC3941251

[ref39] Thabut G. Mal H. (2017). Outcomes after lung transplantation. J. Thorac. Dis. 9, 2684–2691. doi: 10.21037/jtd.2017.07.85, PMID: 28932576 PMC5594127

[ref40] Ulusoy M. Sahin N. H. Erkmen H. (1998). Turkish version of the Beck anxiety inventory: psychometric properties. J. Cogn. Psychother. 12, 163–172.

[ref9001] Wessels‐Bakker M. J. van de Graaf E. A. Kwakkel‐van Erp J. M. Heijerman H. G. Cahn W. Schappin R. (2022). The relation between psychological distress and medication adherence in lung transplant candidates and recipients: A crosssectional study. Journal of Clinical Nursing, 31, 716–725. doi: 10.1111/jocn.15931, PMID: 34216066 PMC9292052

[ref41] Willgoss T. G. Yohannes A. M. (2013). Anxiety disorders in patients with COPD: a systematic review. Respir. Care 58, 858–866. doi: 10.4187/respcare.01862, PMID: 22906542

[ref42] Yusen R. D. Edwards L. B. Dipchand A. I. Goldfarb S. B. Kucheryavaya A. Y. Levvey B. J.. (2016). The registry of the International Society for Heart and Lung Transplantation: thirty-third adult lung and heart–lung transplant report—2016; focus theme: primary diagnostic indications for transplant. J. Heart Lung Transplant. 35, 1170–1184. doi: 10.1016/j.healun.2016.09.001, PMID: 27772669

[ref43] Yusen R. D. Edwards L. B. Kucheryavaya A. Y. Benden C. Dipchand A. I. Dobbels F.. (2014). The registry of the International Society for Heart and Lung Transplantation: thirty-first adult lung and heart–lung transplant report—2014; focus theme: retransplantation. J. Heart Lung Transplant. 33, 1009–1024. doi: 10.1016/j.healun.2014.08.004, PMID: 25242125

